# Supplementary material for: Hydroxytyrosol–Donepezil Hybrids Play a Protective Role in an In Vitro Induced Alzheimer’s Disease Model and in Neuronal Differentiated Human SH-SY5Y Neuroblastoma Cells
Source: Int J Mol Sci. 2023 Aug 30;24(17):13461. doi: 10.3390/ijms241713461 (PMC10488223; doi:10.3390/ijms241713461)
Supplement: Supplementary file 1 [file ijms-24-13461-s001.zip › ijms-2561805-supplementary.pdf]

Supplementary Table 1. Effect of the HT hybrids on acetylcholinesterase, butyrril-cholinesterase and BACE-1 activity.

	**AChE**		**BuChE**		**BACE-1**	
**HT hybrid**	**Concentration (µM)**	**IC_{50} (µM)**	**Concentration (µM)**	**IC_{50} (µM)**	**Concentration (µM)**	**IC_{50} (µM)**
HT1	2.5 – 5.0	6.5 ± 0.8	5 – 10	20.3 ± 2.4	2.5 – 25	11.3 ± 1.2
HT1a	2.5 – 5.0	4.3 ± 0.7	1.5 – 10	4.2 ± 0.4	2.5 – 25	16.9 ± 0.1
HT2	2.5 – 5.0	3.8 ± 0.3	5 – 10	31.8 ± 5.7	2.5 – 25	128.0 ± 42.0
HT3	2.5 – 5.0	4.0 ± 0.4	5 – 10	7.2 ± 0.9	2.5 – 25	76.8 ± 3.0
HT3a	2.5 – 5.0	2.6 ± 0.2	2.5 – 5	4.3 ± 0.9	2.5 – 25	19.7 ± 0.7
HT4	2.5 – 5.0	4.9 ± 0.5	5 – 10	22.9 ± 7.1	2.5 – 25	52.2 ± 2.1
HT4a	2.5 – 5.0	2.9 ± 0.2	2.5 – 5	4.1 ± 0.5	2.5 – 25	115.0 ± 5.1
Donepezil	0.01 – 0.1	0.01 ± 0.005	2.5 – 5	1.3 ± 0.4	0.1 – 0.5	0.18 ± 0.04
Inhibitor I[b]	–	–	–	–	0.5 – 5.5	1.27 ± 0.44
Inhibitor IV[b]	–	–	–	–	0.1 – 0.5	0.12 ± 0.05

[a] C: competitive; NC: non competitive; U: uncompetitive; M: mixed
[b] Known inhibitor of BACE-1 activity
Values are expressed as mean ± SD calculated on three, and four different determinations for ChE, and BACE-1, respectively

Supplementary Table 2. Inhibition by HT hybrids on the Aβ fibril formation.

**HT hybrid**	**Concentration (µM)**	**IC_{50} (µM)**
HT1	20 – 240	375.0 ± 142.8
HT1a	20 – 240	106.4 ± 11.1
HT2	20 – 240	417.6 ± 41.1
HT3	20 – 500	n.d.[a]
HT3a	20 – 500	n.d.[a]
HT4	20 – 500	n.d.[a]
HT4a	20 –	n.d.[a]
Donepezil	20 – 240	183.8 ± 14.7

[a] Inhibition not detectable up to the maximum concentration used

Values are expressed as mean ± SD calculated on three different determinations
